# Editorial: DNA replication barriers and the origins of cancer

**DOI:** 10.3389/fcell.2025.1585382

**Published:** 2025-03-12

**Authors:** Michalis Fragkos

**Affiliations:** Department of Science and Mathematics, School of Liberal Arts and Sciences, American College of Greece, Athens, Greece

**Keywords:** DNA replication, cancer, DNA damage, DNA repair, replication fork stalling

DNA replication is one of the most well-orchestrated cellular processes that needs to proceed in a faithful and timely manner. However, this process is continuously challenged, leading to stalled replication forks that, if not resolved, may activate the process of carcinogenesis. The paper collection of this Research Topic provides insights on the mechanisms that prevent DNA replication blocks, protecting the cell from becoming tumorigenic.

A significant contribution to the understanding of the mechanism that protects cells from replication fork collapse is the work done by the group of Christina Cardoso (Prorok et al.). In this work, scientists set to investigate the potential role of Timeless-Tipin complex in protecting the integrity of the replication fork, in the presence or absence of replication stress induced by either hydroxyurea or aphidicolin. They assayed protein-protein interactions by performing proximity ligation assays and immunostaining with confocal microscopy, using HeLa and HEK293 cells. Given that replication stress leads to the uncoupling of the CMG helicase from DNA polymerase, they were able to observe that Timeless-Tipin complex associates with the helicase complex rather than the PCNA/DNA Polymerase complex. They also found that in both the presence or absence of replication stress, Timeless-Tipin remained associated with chromatin. Another highlight of this study was that, following treatment with either HU or Aphi, the Timeless-Tipin complex interacted with Replication Protein A (RPA), which is a single-stranded DNA binding protein, indicating a potential function of this complex in protecting single-stranded DNA under conditions of replication stress. In conclusion, this study suggests a new role for the Timeless-Tipin complex in contributing to the replication stress signaling response, by interacting with single-stranded DNA via RPA.

Another interesting study reported in this Research Topic of papers was the one by the group of Marta Popovic on the resolution of DNA-protein crosslinks (DPCs) to protect the genomic integrity of the cells (Anticevic et al.). Unresolved DPCs may block DNA replication causing replication fork stalling and collapse, creating double-stranded DNA breaks. This study verified the role of two enzymes of the proteolytic pathway of DPC resolution, called Tyrosyl-DNA phosphodiesterase 1 (TDP1) and 2 (TDP2), by using zebrafish as a model organism. Initially, they identified the two *tdp2* orthologs in zebrafish, *tdp2a* and *tdp2b*, and, by performing qPCR, revealed gender-specific gene expression. In specific, *tdp2a* was expressed in the testes, while tpd2b was expressed in the ovaries. Both genes were equally expressed in different adult tissues, such as brain, kidney and intestine, whereas only *tdp2b* was abundantly expressed in the zebrafish embryos. They then knocked down the two genes using morpholino oligonucleotides and found that inactivation of *tdp2b* only is sufficient to impair TDP2 activity, leading to accumulation of DPCs and consequent genomic instability. Quantification of DPCs was performed using the RADAR (Rapid Approach to DNA Adduct Recovery) assay, whereas genomic instability was assessed by quantifying γH2AX phosphorylation via Western blotting. In summary, the above work showcases the important role of TDP2 in preventing double strand DNA breaks induced by DNA-protein crosslinks that, amongst other sources, might come as a result of replication stalling.

A different study by Yoshizawa et al., who set to understand the cause of instability observed in mammalian haploid somatic cells, is also reported in this Research Topic of papers (Yoshizawa et al.). Haploid cells may arise as a result of tumorigenesis, leading to further genomic instability. Haploid cells may then undergo spontaneous genome duplication, converting them to diploid. Haploid cell instability often comes as a result of the uncoupling between DNA replication and centrosome duplication cycles. To evaluate this hypothesis, Yoshizawa et al. extended the cell cycle of the human haploid cell line HAP1, by performing three rounds of thymidine blocking and investigated whether extension of the G1/S phase and consequent coupling with the centrosome duplication cycle would affect haploid instability. Their result showed that this recoupling of the DNA replication and centrosome duplication cycles was indeed effective in maintaining genomic stability in haploid cells. Moreover, inhibition of cdk2, a cell-cycle regulator that controls the coupling between DNA replication and centrosome duplication, in thymidine-treated haploid cells, reinduced genomic instability in haploid cells. Overall, these data show that DNA replication-centrosome duplication coupling, and not simply an extension of the S-phase, is a prerequisite for allowing haploid cells to maintain genomic stability.

DNA replication blocking can become one of the causes of DNA damage in mammalian cells. Replication of DNA can be stalled by various agents, including Reactive Oxygen Species (ROS), causing severe genomic instability that may lead to cancer. The work by Li et al. shows that ROS may cause DNA breaks in high glucose (HG)-treated Human Kidney-2 (HK2) cells, resulting in activation of a sensor of double strand DNA breaks called AIM2 (Absent In Melanoma 2; Li et al.). Their study showed that activated AIM2 induces an inflammasome-mediated type of programmed cell death called pyroptosis. The results of this work may explain the elevated levels of AIM2 in proximal tubular epithelial cells of patients suffering from diabetic nephropathy (DN). Taken together, this study shows a link between ROS-induced double-stranded DNA breaks and pyroptosis, both in cell lines or patient cells, providing new insights for the potential use of AIM2 as a therapeutic agent for DN.

Taken together, this Research Topic of papers, even though quite diverse, provides new insights in the field of molecular carcinogenesis and the role of DNA replication stress in this process. Studying the mechanism by which cells resolve replication stalled forks, so as to prevent the accumulation of DNA damage, will continue being a target for understanding the molecular basis of cancer, but also for developing new therapies to treat this disease.

